# Effect of the environment on gait and gaze behavior in older adult fallers compared to older adult non-fallers

**DOI:** 10.1371/journal.pone.0230479

**Published:** 2020-03-20

**Authors:** Lisa A. Zukowski, Gözde Iyigün, Carol A. Giuliani, Prudence Plummer

**Affiliations:** 1 Department of Physical Therapy, High Point University, High Point, North Carolina, United States of America; 2 Department of Physiotherapy and Rehabilitation, Faculty of Health Sciences, Eastern Mediterranean University, Famagusta, North Cyprus, Turkey; 3 Division of Physical Therapy, Department of Allied Health Sciences, University of North Carolina at Chapel Hill, Chapel Hill, North Carolina, United States of America; 4 Human Movement Science Curriculum, University of North Carolina at Chapel Hill, Chapel Hill, North Carolina, United States of America; University of Rochester, UNITED STATES

## Abstract

**Introduction:**

Compared to controlled laboratory settings, the real world is highly distracting with constant demands on visual attention to avoid hazards and falling. Fall-risk assessments do not adequately take into account the potential role of everyday distractions and environmental hazards. The purpose of this project was to identify the effect of the environment on gait and gaze behavior during walking in older adult fallers relative to non-fallers.

**Methods:**

Thirteen older adult fallers (76.8±9.4 years, 3.2±2.3 falls in last year) and 13 age-matched non-fallers (78.3±7.3 years, 0 falls in last year) participated. Participants walked in a real-world and lab setting while gait and gaze were recorded. Gait variables were stride length variability, stride duration variability, and stride velocity. Gaze was analyzed for percentage of time fixating and average fixation duration coded across 6 areas of interest (AOIs) in the visual surroundings.

**Results:**

Non-fallers walked faster than fallers, but there were no other group or environment effects on gait. For gaze behavior, fallers had shorter fixation durations on the near environment than non-fallers, but only in the real world. In the real world relative to the lab, fallers decreased fixation durations on the near environment but increased durations on near people. In the real world, participants spent a greater proportion of time fixating on people than on the walking path or the near environment compared to the lab. After adjusting for baseline gait speed, fallers had shorter fixation durations than non-fallers in both environments.

**Conclusions:**

Our results indicate that in a busy environment, older adults concentrate most of their overt visual attention on people when navigating their walking path. Fallers in particular focus longer on people near to them and have overall shorter fixations than non-fallers. Visual focus while walking in a busy environment should be further explored as a fall-risk factor.

## Introduction

Our everyday world, hereafter referred to as the “real world”, is far more complex and unpredictable than a laboratory or clinic where mobility in healthy, older adults is traditionally evaluated and fall risk factors are identified. Indeed, the real world exposes us to environmental hazards, such as pedestrian and vehicular traffic, pets, uneven terrain, and obstacles like garden debris that must be avoided while walking. Not surprisingly then, these complex and unpredictable environmental hazards have been identified as a major cause of falls in healthy, older adults [1]. Yet, traditional fall-risk assessments do not adequately take into account the role of distractions and hazards experienced in everyday life. Therefore, it is of great importance to understand the underlying factors contributing to falls among older adults in everyday life so that we can design more effective fall prevention programs that address real-world fall risk factors.

Compared to the low-distraction setting of a laboratory, the real world is a highly distracting environment with constant visual as well as gait adaptability demands to negotiate hazards and avoid collisions or loss of balance. However, very few studies have examined how walking and gaze behavior may differ in the real world compared to a laboratory setting. A previous study of young adults comparing walking in real-world and lab environments showed that individuals fixate more on the walking path in the real world, whereas they fixate on distant objects when watching a first-person viewpoint video of the same task [2]. We also know that young adults walk faster in an indoor real-world setting than they do in a lab environment [3]. However, when walking outside in natural terrain, as the terrain becomes rougher, young adults increasingly focus their gaze on the upcoming path, walk more slowly, and make more walking trajectory deviations to find good footholds [4]. Regardless of the difficulty of the terrain, individuals tend to focus their gaze roughly 1.5 s ahead of their current position, indicating a limitation of visual memory in determining walking trajectory and control of foot placement [4]. Taken together, the results of these studies suggest the environment affecting gait and gaze behavior, with greater environmental complexity and unpredictability likely resulting in more frequent fixations on the walking path and greater gait variability, as individuals try to avoid objects and pedestrians.

We currently know very little about how gait and gaze performance differ between real-world and lab environments in older adults, yet the differences between these environments are likely to be more apparent in older adults because of a relative increase in demands associated with age-related declines. Relative to the laboratory, the difference in gait and gaze performance in the real world could have the potential to impact walking safety and fall risk and thus warrants examination to determine if changes to fall prevention training to address these differences could further increase the effectiveness of fall prevention programs. Specifically, walking in a real-world environment should place high demands on visual attention and gait adaptability (i.e., adapting gait to meet environment demands), both of which have been shown to decline with age [5–7]. Age-related declines in visual attention encompass a decrease in processing speed and difficulty with selective attention that could result in less active scanning of the relevant environmental cues and longer fixation durations on certain people or objects in the more complex real-world environment [7]. These declines in visual attention and potential changes in gaze behavior are significant because they are compounded by age-related declines in spatial working memory that impact visuomotor adaptation [8] and have been linked to an increased likelihood of collision with a car during a street crossing paradigm [7]. These changes similarly increase the likelihood of an older adult walking in a real-world environment colliding with someone or something and potentially falling. Age-related declines in gait adaptability include increased gait variability while simultaneously walking and performing a cognitive task [5] and while walking with concurrent visual distracters [6]. Together with declines in visual attention and visuomotor adaptation, these results suggest that older adults experience a relative increase in demands due to age-related declines and may have a reduced ability to quickly and efficiently respond to the unpredictable demands of the real world. Since age-related declines in visual attention and gait adaptability are greater in older adults fallers than non-fallers [9–13], environmental differences in gait and gaze performance may also be exacerbated in older adult fallers relative to non-fallers.

Identifying differences in gait and gaze behavior between older adults with and without history of falling, as well as any influences of the environment on faller and non-faller differences may improve our understanding of factors contributing to falls. The purpose of this project was to compare the effect of environment (low versus high distraction) on gait parameters and gaze behavior (overt visual attention) during walking in older adult fallers and non-fallers. We hypothesized that we would observe 1) an environment effect, such that relative to the lab, in the real world older adults (fallers and non-fallers) would exhibit greater gait variability, longer average fixation durations, indicative of less active scanning or acquiring and storing more visual information about particular objects, and a greater percentage of fixations on the travel path, indicative of prioritizing optimal foot placement and safe navigation through a busy environment; and 2) the effect of environment to be greater on gait parameters and gaze behavior (as identified in the first hypothesis) in fallers than non-fallers.

## Methods

### Participants

Participants were older adult fallers, at least 60 years old reporting two or more falls within the past 12 months, and were recruited from the local community. A fall was operationally defined as “an unexpected event in which the participants come to rest on the ground, floor, or lower level” [14]. Non-faller comparison participants, defined as having reported no falls in the last 12 months, were also recruited from the local community. Non-fallers were recruited to match each enrolled faller on age (± 5 years), gender, and education (± 2 years). Age and gender were matched because increasing age and being female are associated with a higher risk of falling [15]. We controlled for years of education because more education corresponds to higher cognitive functioning [16], the latter of which is related to reduced risk of falling [17]. In addition to being at least 60 years old, all participants also had to be able to walk continuously for 3 minutes either with or without an assistive device, communicate verbally in English, be able to follow a 3-step command, have no orthopedic problem or pain affecting gait, no significant hearing or vision impairments, and no pre-existing neurological disorders. We did not exclude individuals who used an assistive device because many older adults use an assistive device to compensate for poor balance control. Thus, excluding them would bias our sample of fallers to only those with minimal balance control deficits, unacceptably limiting the external validity and usefulness of the findings. Only 2 of the 13 fallers and none of the non-fallers used an assistive device. The study was approved by the University of North Carolina at Chapel Hill Institutional Review Board, and all participants provided informed consent before completing any study procedures.

The study was powered to detect an Environment x Group interaction effect size of f = 0.32, which was based on estimates of gait variability in older adults during normal, cognitively challenging, and visually perturbed walking tasks [18], ranging from 2.9% to 6.7%. In the absence of gait variability estimates among older adults during real-world walking, these previously reported data in cognitive and visually demanding walking tasks [18] were deemed the best available estimates of effect size and variance for our power analysis. Assuming a SD of 2.3% for differences in gait variability and a correlation among repeated measures of 0.5, α = 0.05, and power = 90%, the total number of participants needed was 28 (14 fallers and 14 non-fallers).

### Procedures

Procedures were conducted over two separate testing sessions held no more than a week apart. In the first testing session, a battery of assessments was performed to evaluate cognition, functional mobility, vision, physical activity, and balance self-efficacy, all of which have been shown to relate to fall risk. Cognitive and language abilities were assessed using the Montreal Cognitive Assessment [19], WAIS Vocabulary subtest [20], WAIS Digit Symbol Substitution and Copy subtests [20], Comprehensive Trail Making Test (CTMT) [21], and a computerized Stroop color-word interference test. Functional mobility was assessed using the 10 Meter Walk Test, Timed Up and Go test (TUG) [22], Four Square Step Test [23], and Dynamic Gait Index [24]. Vision was assessed using the Snellen visual acuity test and the Melbourne Edge Test of contrast sensitivity [25]. Finally, community participation and self-efficacy were assessed with the Physical Activity Scale for the Elderly [26] and the Activities-specific Balance Confidence Scale [27]. Demographic information were also collected.

In the second testing session, participants performed two walking trials in both a real-world environment (busy hospital lobby) and a laboratory setting, for a total of four walking trials. Two walking trials were performed in each environment to try to capture average walking performances and diminish the impact of any random occurrences or chance incidences. The order of environments was randomized across participants but case-controlled between the faller and non-faller groups. For each walking trial, participants walked continuously at their self-selected speed for 1 minute along a length of 30 m, with ample room for turning at either end. We recorded gait (stride data) during each 1-minute trial using the 5-sensor LEGSys+ wireless, tri-axial accelerometer and gyroscope system (100 Hz, Biosensics, Cambridge, MA). The five sensors were attached to each shin, each thigh, and the low back. Gaze data were recorded using the SMI Eye Tracking Glasses 2 Wireless (SensoMotoric Instruments, Boston, MA), which collected binocular gaze orientation (60 Hz) and a digital video of the scene view (30 Hz) taken from the nose bridge of the glasses during each walking task. The surrounding environment, which included people and objects that were both within and outside of the participant’s walking path and/or view, was recorded with a video camera during each walk. All of the people in each environment were there of their own accord and did not receive any instructional set or directions from us. Additionally, the hospital lobby used as the real-world environment formed the intersection of three different hospitals within the medical center, ensuring that a number of people were seated in the environment or walking in all directions throughout the day. Scheduling was arranged to avoid days of the week and times of day that the hospital lobby was either very busy or unusually quiet.

### Data processing

For each 1-minute walking trial, all of the individual stride values for stride length, stride duration, and stride velocity were recorded, and then the mean value and standard deviation for stride length, stride duration, and stride velocity were computed with a custom Matlab (MathWorks, Natick, MA) program that utilized the LEGSys™ algorithm, which has been validated in an older adult population [28]. Participants took, on average, 53 (SD 4.1) strides per walking trial (including turns). In order to examine gait that is typical of everyday walking, turning strides at either end of the walkway were included with strides recorded while walking along the mostly straight path in each environment. The first stride was removed if it was less than half the magnitude of the second stride, and the last 3 strides of walking data were removed, but all other strides that were recorded during spontaneous acceleration or deceleration were included in the within-trial mean and variability values. The three gait dependent variables were stride length variability (stride-to-stride coefficient of variation [CV], %), stride duration variability (stride-to-stride CV, %), and average stride velocity (m/s). For each of the 3 dependent variables, we averaged the values from each of the 2 trials and used the average for each environment in the analysis.

**Fig 1:**
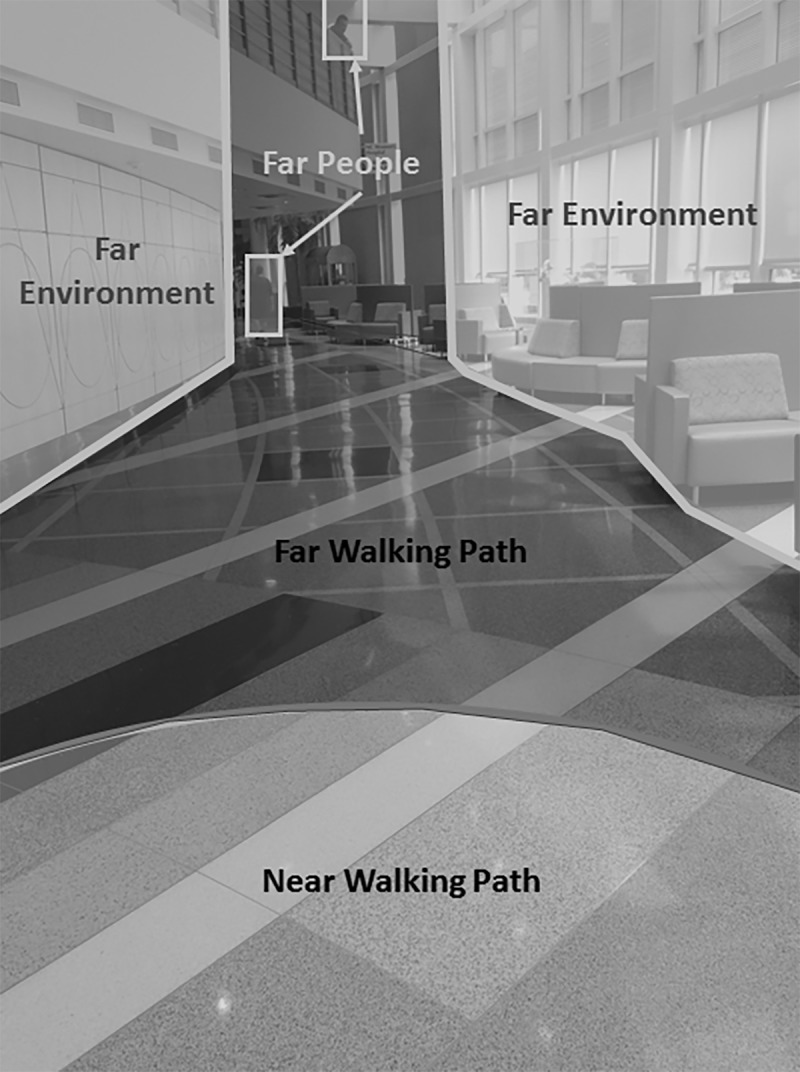
Snapshot of a portion of the real-world environment with 4 of the 6 AOIs demarcated. This photo does not depict near people (in order to protect the privacy of those visiting the hospital) or near environment AOIs, but it provides an illustration of how the walking path, environment and people were defined, as well as it illustrates how they were further distinguished as near or far.

Using BeGaze software (SensoMotoric Instruments, Boston, MA), gaze orientation was designated by a cursor and superimposed on top of the scene view. This video was used to categorize each of the participants’ fixations during each walking trial as being focused on one of six pre-defined areas of interest (AOIs) in the participants’ field of view. The six AOIs were: walking path, people, and the surrounding environment, each further categorized as either near or far from the participant (Fig 1). Near AOIs were defined as path/people/environment within 4–6 steps of the participant, whereas far AOIs were path/people/environment estimated to be beyond 4–6 steps. Because participants were free to move their heads as they walked in each environment, the perspective of the scene view video (e.g. looking straight ahead, looking down at the floor, looking to the left of the walking trajectory, etc.) changed frequently throughout each trial. The constantly changing perspective made it difficult to determine an exact distance (i.e., 6 steps) between the participant and an item/landmark/person, but using environmental clues within the video scene, the rater could determine, with some degree of consistency, if a point of fixation was close enough to the participant that if they walked towards it, it would be in their personal space, defined as 1.2 meters by Hall et al. [29], within the next few steps, which was the basis for the 4–6 steps “near” designation. Walking path AOIs were any part of the participant’s walking trajectory, excluding people. People AOIs referred to any person who was standing, walking, or sitting in the participant’s field of view, in or outside the walking path. People were defined separately from the walking path and surrounding environment because they move and thus could attract/distract visual attention. Environment AOIs were all non-people objects outside the person’s walking path. The six AOIs, based upon categories originally defined by Foulsham et al. [2], were chosen because they succinctly represent the range of objects fixated upon in an environment, both relevant and irrelevant to avoiding collisions and walking safely. The same rater categorized each of the fixations during each trial in order to maximize the consistency of fixation categorization across participants and trials.

The two dependent variables for the analysis of gaze behavior were percentage of time fixating, out of the total trial duration, and average fixation duration (ms). For each of these dependent variables, we computed the average for each AOI across the 2 walking trials in each environment. Fixations shorter than 200 ms were excluded from the analysis because it has been previously established that fixations during visual search and scene viewing are at least 200 ms in duration [30].

From the video recordings, we quantified the environmental busyness during each trial by manually counting the people present. Busyness was defined as the number of people that were observed to be in the participant’s field of view throughout each trial, including both people inside and outside of the participant’s walking path but did not include people outside of the participant’s view (e.g., behind the participant). We averaged the busyness (the person count) across the two trials in each environment for analysis.

### Statistical analysis

Independent samples t-tests were used to compare faller and non-faller groups in terms of age, gender, years of education, and other cognitive and mobility performance measures. A paired samples t-test was used to compare the environmental busyness of the lab relative to the lobby, and independent samples t-tests compared the environmental busyness between fallers and non-fallers in each testing environment. A Group (faller, non-faller) x Environment (lab, lobby) repeated measures ANOVA was used to examine the effects of group and environment and their interaction on gait variables. However, in light of observed between-group differences in cognition and mobility, we conducted a follow up repeated measures ANCOVA with TUG and CTMT scores as covariates to determine the effects on gait variables when controlling for these differences. In a secondary analysis, the relationship between environmental busyness (person count) in each environment and changes in gait performance (unadjusted values from the lab to the real world) was examined using Spearman’s rho (r_s_) correlation coefficients to explore whether environment-related changes in gait were associated with how many people there were in each environment. To analyze gaze variables, we used a Group x Environment x AOI repeated measures ANOVA. Again, a follow-up ANCOVA with TUG and CTMT scores as covariates was used to examine effects when adjusting for group differences in cognition and mobility. Two-way ANOVA/ANCOVA analyses were used to follow up significant three-way interactions, and Bonferroni post hoc tests were used to examine significant interaction effects as needed. All statistical procedures were performed using SPSS 25 and α = 0.05.

## Results

### Characteristics of participants and environments

Fifteen fallers and 15 non-fallers participated in the study, but technical difficulties during data collection resulted in a loss of data from 2 fallers and 2 non-fallers. The analyzed sample included 13 older adult fallers (76.8±9.4 years, 10 females) and 13 older adult non-fallers (78.3±7.3 years, 9 females). As expected, there was a significant difference in reported fall history between the fallers and non-fallers, but there were no significant differences between the groups in the matched variables (age, gender, education) (Table 1). The groups also differed on several aspects of cognition and all measures of mobility (Table 1). These differences between fallers and non-fallers are consistent with previous studies [23,24,31–33].

**Table 1:** Results of independent samples t-tests comparing demographic characteristics, cognitive, functional mobility, and vision assessments, and self-reported community participation of fallers and non-fallers.

	Fallers (n = 13)	Non-fallers (n = 13)	p-value
Demographic Characteristics			
Age (years)	76.8±9.4	78.3±7.3	p = 0.65
Gender	3 males, 10 females	4 males, 9 females	p = 0.67
Years of Education	16.8±3.3	16.7±2.3	p = 0.87
Number of Falls in Last 12 Months	2 (2–4)	0 (0–0)	**p<0.001**
**Cognitive Assessments**			
Montreal Cognitive Assessment (max. 30)	26.2±2.7	26.9±2.3	p = 0.54
WAIS Vocabulary Subtest (max. 70)	56 (54–60)	59 (56–62)	p = 0.18
WAIS Digit Symbol Substitution Subtest (max. 93)	40.1±9.3	51.0±6.5	**p = 0.002**
WAIS Digit Symbol Copy Subtest (sec)	89.9 (80.9–115.6)	76.3 (69.4–88.4)	**p = 0.03**
Comprehensive Trail Making Test Composite Index	43.9±7.1	54.3±5.6	**p<0.001**
Stroop Color-word Test Interference Reaction Time (ms)	455.2±137.3	333.4±98.1	**p = 0.02**
**Functional Mobility Assessments**			
10 Meter Walk Test (m/s)	1.06±0.27	1.27±0.10	**p = 0.02**
Timed Up and Go (sec)	9.8 (8.4–13.4)	8.0 (7.4–8.5)	**p = 0.004**
Four Square Step Test (sec)	13.0 (7.6–17.1)	8.9 (8.7–9.6)	**p = 0.02**
Dynamic Gait Index (max. 24)	20 (17–22)	23 (22–24)	**p = 0.003**
**Vision Assessments**			
Snellen Vision Acuity (normal is 20/20)	20/40 (20/70–20/25)	20/20 (20/40–20/20)	**p = 0.04**
Melbourne Edge Test of Contrast Sensitivity (max. 24)	19 (19–20)	20 (19–21)	p = 0.20
**Community Participation and Self-Efficacy**			
Physical Activity Scale for the Elderly	132.9±61.3	140.8±78.0	p = 0.78
Activities-Specific Balance Confidence Scale (max. 100)	83.1 (56.4–87.8)	90.6 (87.4–97.2)	**p = 0.009**

Values are Mean±SD or Median(IQR).

The real-world environment was significantly busier than the lab setting in terms of people encountered during the walking trials (Table 2). However, there were no significant differences in busyness in the environments experienced by fallers and non-fallers (Table 2) and, thus, on average, all participants were presented with similarly quiet lab and similarly busy real-world environments to navigate.

**Table 2:** Paired samples t-test comparing the busyness of the environments across fall status and independent samples t-tests comparing environmental busyness between fallers and non-fallers in each environment.

	Environmental Busyness (number of individuals)		p-value
**Lab** (n = 26)	1.1±1.0		**p<0.001**
**Real World** (n = 26)	22.4±7.7		
	Environmental Busyness (number of individuals)		p-value
	**Fallers** (n = 13)	**Non-fallers** (n = 13)	
**Lab**	1.1±1.0	1.1±1.0	p = 1.00
**Real World**	21.8±8.7	23.0±7.0	p = 0.70

Values are Mean±SD.

### Gait variables

The Group x Environment ANOVA exhibited only a significant main effect of Group on gait speed (F(1,24) = 5.45, p = 0.03, $\eta_{p}^{2}$= 0.185), such that, on average, non-fallers walked 0.2 m/s faster than fallers (Table 3). The main effect of Group on gait speed was not significant (F(1,22) = 0.032, p = 0.859, $\eta_{p}^{2}$= 0.001) after adjusting for TUG and CTMT. The covariate, TUG, was significantly related to gait speed (F(1,22) = 11.569, p = 0.003, $\eta_{p}^{2}$= 0.345). There was no significant Group x Environment interaction effect on gait speed regardless of whether the analysis controlled for group differences in cognition and mobility (Table 3). None of the effects on gait variability were significant with either the ANOVA or ANCOVA (Table 3).

**Table 3:** Average walking performance for fallers and non-fallers in the lab and real-world environments.

	Fallers (n = 13)	Non-fallers (n = 13)	Group x Environment	
			p	$\eta_{p}^{2}$
**Stride Velocity (m/s)**				
Lab	1.11±0.24	1.29±0.14	0.631	0.010
Real World	1.13±0.23	1.30±0.15		
**Stride Length CV (%)**				
Lab	11.47±5.00	9.41±2.61	0.389	0.031
Real World	11.71±3.50	10.87±2.67		
**Stride Duration CV (%)**				
Lab	6.07±4.16	6.48±2.81	0.863	0.001
Real World	6.27±3.51	6.97±4.09		

Values are unadjusted Mean±SD.

The number of people present in the real-world environment, which includes both individuals in and outside of the participant’s walking path, was related to the change between the lab and lobby in gait speed (r_s_ = 0.56, p = 0.003) and stride length variability (r_s_ = -0.56, p = 0.003), across fallers and non-fallers. The environment-related change in gait speed and stride length variability tended to be greater when there were more people in the real world. Upon further exploration, it was determined that the relationship between environmental busyness and environmental changes in unadjusted gait speed and stride length variability was driven by the number of people (bystanders) in the real-world environment who were outside the participant’s walking path (21.65±7.47 people, r_s_ = 0.58, p = 0.002 and r_s_ = -0.54, p = 0.005 for gait speed and stride length variability, respectively) rather than by people within the participant’s walking path (0.75±0.72 people, r_s_ = -0.07, p>0.05 and r_s_ = -0.27, p>0.05 for gait speed and stride length variability, respectively). The lack of a relationship between the number of people in the participant’s walking path and environment-related changes in gait may be a consequence of very few people being directly in the participant’s walking path. There was no relationship between the environmental busyness of the real world and environment-related changes in unadjusted stride duration variability or between environmental busyness of the lab and changes in unadjusted gait variables (all p>0.05).

### Gaze variables

**Fig 2:**
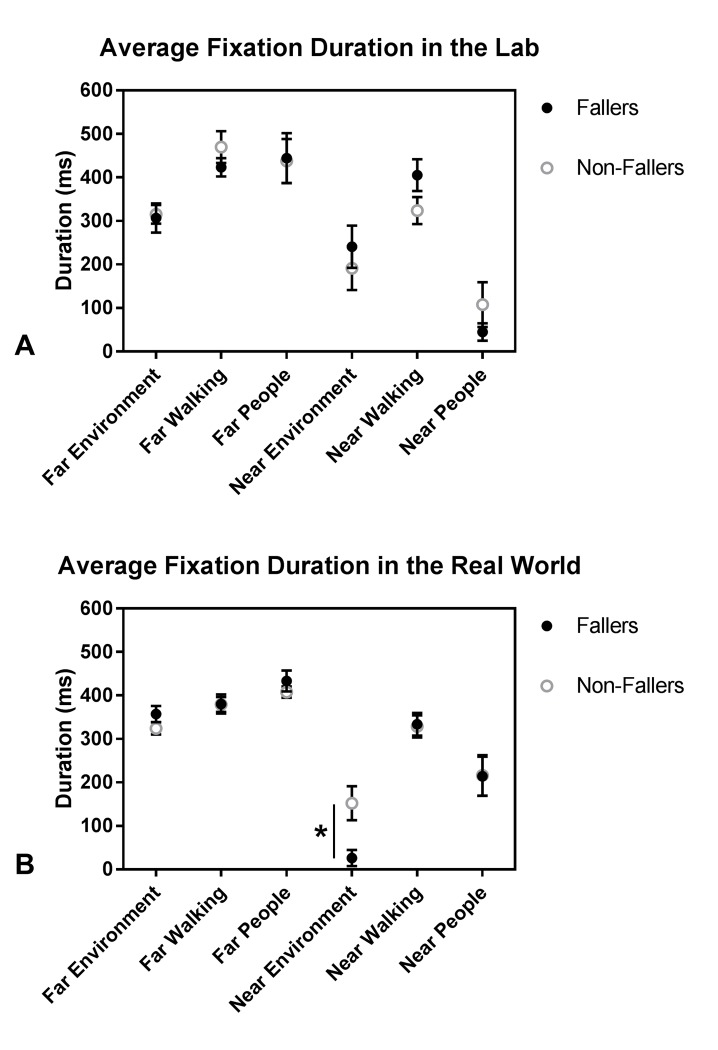
Average fixation duration as a function of the 6 AOIs and fall status in A) the Lab environment, and B) the Real World environment. The Group x AOI interaction was significant in the real world but not the lab; significant differences are indicated (*). Values are unadjusted Mean±SEM. Some of the average fixation duration values are less than 200 ms because the data in the figures represent the average across the 2 trials and across participants. These average values include fixation durations of 0 ms when participants never fixated on a particular AOI for at least 200 ms in a trial.

**Fig 3:**
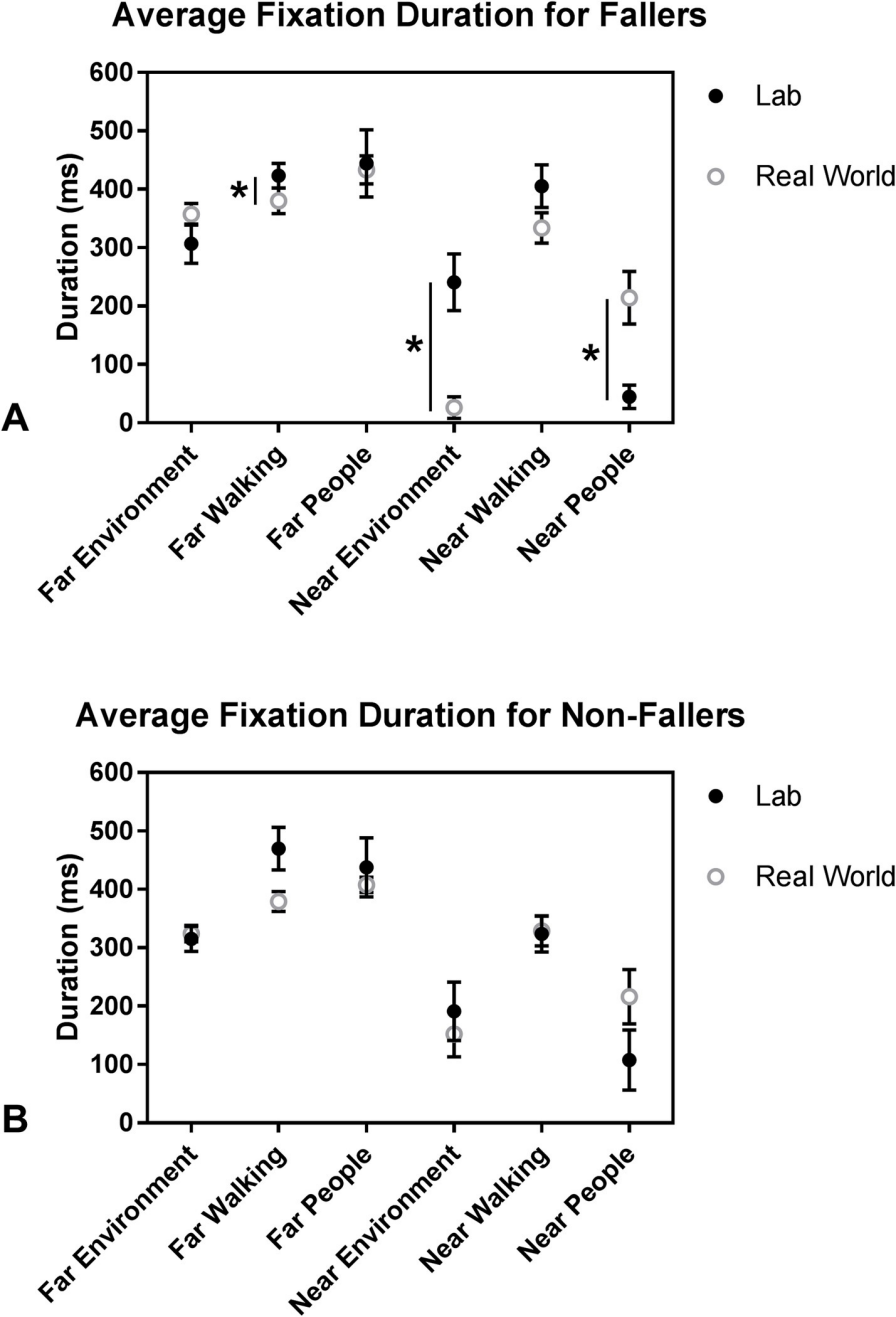
Average fixation duration as a function of the 6 AOIs and environment for A) Fallers, and B) Non-Fallers. The Environment x AOI interaction was significant for fallers but not non-fallers; the significant differences are indicated (*). Values are unadjusted Mean±SEM. Some of the average fixation duration values are less than 200 ms because the data in the figures represent the average across the 2 trials and across participants. These average values include durations of 0 ms when participants never fixated on a particular AOI for at least 200 ms in a trial.

The Group x Environment x AOI ANOVA revealed a significant 3-way interaction effect for average fixation duration (F(3.59,86.21) = 2.505, p = 0.05, $\eta_{p}^{2}$= 0.095). To investigate the 3-way interaction, the data were split by Environment and by Group, separately. It was determined that the 3-way interaction occurred because the Group x AOI interaction was significant in the real world but not in the lab (Fig 2), and the Environment x AOI interaction was significant for fallers but not non-fallers (Fig 3). Bonferroni post hoc tests of the follow-up two-way ANOVA analyses revealed that in the real world, fallers had significantly shorter average fixation durations on the near environment than non-fallers (Fig 2B); there were no other Group effects for any other AOIs in the lobby. Further, among fallers, average fixation duration was longer in the lab than the real world for near environment and the far walking path but shorter in the lab than the real world for near people (Fig 3A). Average fixation duration across AOIs was not influenced by environment in non-fallers (Fig 3B).

After adjusting for TUG and CTMT scores, the Group x Environment x AOI ANCOVA still revealed a significant 3-way interaction effect for average fixation duration (F(5,110) = 2.759, p = 0.02, $\eta_{p}^{2}$= 0.111). The covariate, TUG, had a significant effect on average fixation duration (F(1,22) = 7.709, p = 0.011, $\eta_{p}^{2}$ = 0.259). To investigate the 3-way interaction, the data were split by Group and by Environment, separately. The significant 3-way interaction effect occurred because the Group x AOI interaction was significant in the lab but not in the real world, which is the reverse finding of the ANOVA. There was also a significant effect of Group in the real world but not in the lab. The Environment x AOI interaction was no longer significant, nor were there any other significant main effects of Environment or AOI. TUG had a significant effect on average fixation duration for fallers but not non-fallers. Bonferroni post hoc tests of the follow-up two-way interactions revealed that in the lab, fallers had significantly shorter average fixation durations on the far and near walking path and far people than non-fallers. Additionally, in the real world, fallers had significantly shorter average fixation durations than non-fallers.

**Fig 4:**
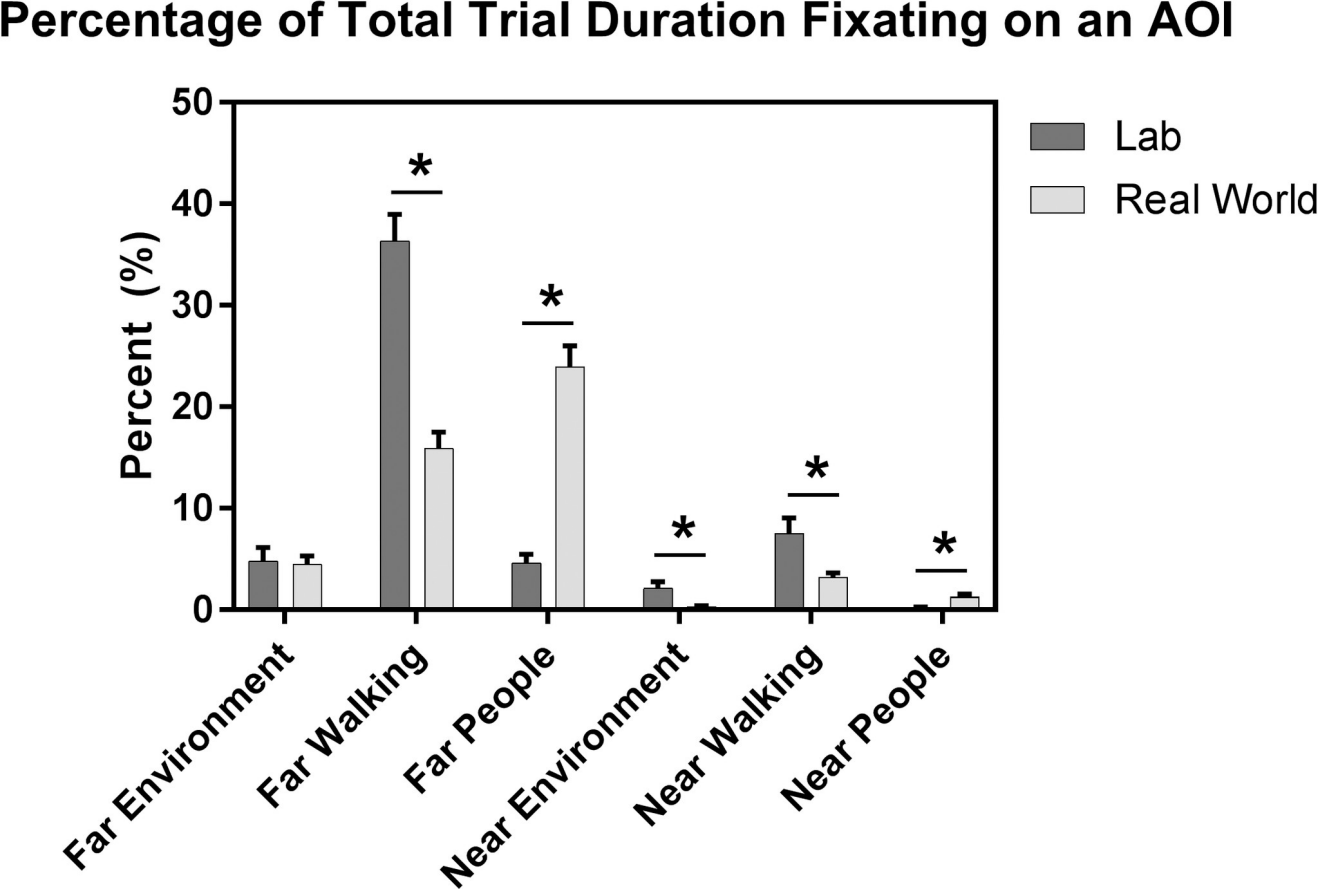
Percentage of total trial duration fixating as a function of each of the 6 AOIs and the 2 environments. Significant differences are indicated (*). Values are unadjusted Mean±SEM.

For percentage of time fixating, the ANOVA revealed a significant Environment x AOI interaction (F(2.27,54.42) = 60.494, p<0.001, $\eta_{p}^{2}$ = 0.716). Specifically, participants fixated on the walking path AOIs (near and far) and near environment for more of the trial in the lab than in the real world; whereas they fixated on people AOIs (near and far) for a greater percentage of time in the real world than in the lab (Fig 4). There was no difference between the real world and the lab on percentage of time fixating on the far environment (Fig 4). After adjusting for TUG and CTMT, there was still no significant 3-way interaction effect, but there was also no significant effect of either covariate on percentage of time fixating, so the prior results should be retained and interpreted.

## Discussion

The purpose of this study was to determine the effect of the environment on gait and gaze behavior in older adults and to identify performance differences between older adult fallers and older adult non-fallers. In terms of gait performance, we did not observe significant differences in gait speed or gait variability between the environments. However, the presence of a weak relationship between the change in gait speed and stride length variability from the lab to the real world and the number of people in the real-world environment provides some evidence that higher person volume in the environment may be the environmental feature that drives gait speed and stride length variability. This finding partially supports our hypothesis because, although gait did not change in a systematic pattern from the lab to the real world settings, the observed changes in gait may have still been influenced by the busyness of the real world environment. These results are in contrast with previous research that observed young adults to consistently walk faster in an indoor real-world environment than they do in a lab setting [3] but in agreement with previous research that observed young adults to walk slower in natural terrain as it became more complex [4]. However, it is difficult to specifically compare our gait results, which included strides during relatively straight path walking as well as during turns, to the gait reported in previous literature, which excluded turning. The results of the present study are, however, in agreement with previous literature demonstrating that, aging can result in a reduced ability to ignore distractions [34], such as people, and visual distractions can cause older adults to walk more slowly and with increased gait variability, as compared to walking without visual distractions [6]. Exploring environmental busyness as a possible moderator between the effect of environment and changes in gait may be an interesting focus for future research.

In addition to the weak environmental influence on gait speed for all older adults, non-fallers walked faster than fallers in both environments. This is consistent with previous research that shows that fall history is associated with slower walking speeds [31]. After adjusting for baseline performance on the TUG, there was no longer a difference in gait speed between fallers and non-fallers in either environment. These adjusted results are not surprising because the TUG incorporates a measure of gait speed, so it is logical that after controlling for baseline TUG differences across participants, the difference between the groups in gait speed disappears. It is, however, surprising that there was no difference in gait variability between fallers and non-fallers in either environment. The lack of a difference may be explained by the already high gait variability exhibited by non-fallers in this study, which was similar in magnitude to that exhibited by fallers. Hausdorff et al. [9] reported stride duration CV values of 2.4% for older adult non-fallers and 4.1% for older adult fallers during a 6-minute walk test, both of which are much smaller in magnitude than the 6.1% for non-fallers and 6.5% for fallers calculated from the lab setting conditions in the present study. Differences between these two sets of results may be a consequence of different protocols. Specifically, the Hausdorff et al. [9] study had participants walk around a large circular path, instructed participants to not stop unless absolutely necessary, and removed the first and last 15 seconds of walking data; whereas the current study had participants walk along a mostly straight path with room to make wide turns at either end, instructed participants to walk as they normally would during everyday activities, and only removed the first stride (when less than half the magnitude of the second stride) and last 3 strides of walking data. Thus the gait variability measures of the present study are most likely higher because participants were accelerating and decelerating to avoid bystanders and to turn. Thus, we believe the estimates of stride variability observed here are more likely than previous studies to represent true gait variability in typical everyday walking. This study provides additional new information to suggest that gait variability typical of everyday walking does not appear to differ between community-dwelling fallers (i.e., fallers who were not highly balanced impaired) and non-fallers in either environment.

In contrast to our hypothesis, we did not observe an overall increase in average fixation durations in the real world or any main effect of environment on average fixation duration on the different AOIs. This failure to observe a difference between the two environments is relevant because visual information is only acquired and stored in memory during fixations, not during saccades [35]. Thus, older adults did not appear to exhibit a change in active scanning or in their strategy of visual information processing in the real world [36].

We predicted that older adults would fixate more frequently on the travel path in the real world, compared to the lab, as a strategy for optimal foot placement and safe navigation through a busy environment. However, in contrast to our hypothesis, older adults increased the frequency with which they fixated on near and far people and decreased the frequency with which they fixated on the near and far walking path in the real world compared to the lab. In the real world, the far and near walking path were still the second and fourth, respectively, most frequently fixated upon AOIs, but far people was the most frequently fixated upon AOI, with near people being the fifth (Fig 4). Thus, older adults spent a large proportion of their time fixating on the walking path in the real world, but they still reduced the frequency with which they fixated on the walking path AOIs and increased the frequency with which they fixated on people. This change in gaze behavior may have been in part due to the greater overall number of people in the real world, as compared to the lab. However, it may also reflect that people within and adjacent to the walking path in the real world were more salient as environmental hazards than fixating on the walking path for proper foot placement and selecting a safe walking path. Participants may have needed longer fixation time on the people in their environment to fully process their location or observe them for possible movement into the walking path and potentially come up with a motor plan to avoid them. This explanation is supported by the work of Di Fabio et al. [37], which illustrated that typical age-related slowing of central processing resulted in longer fixation times on hazards within the walking path. People adjacent to the walking path may have still been relevant by offering helpful auditory or visual cues to safely navigate through the lobby. Alternatively, participants may have increased their proportion of fixation on people because they were a source of distraction, preventing older adults from focusing on their walking and proper foot placement or walking path, and as previously mentioned, older adults have a reduced ability to ignore distractions [34].

Although, generally, the older adults in this study fixated on people more frequently than on the walking path and near environment in the real-world setting as compared to the lab, non-fallers fixated on the near environment for longer average durations than fallers in the real world environment. These results partially corroborate our hypothesis that the environmental influence would be greater on fallers than non-fallers. While fallers may have been less comfortable in diverting attention from urgent environmental hazards in the lobby, such as people and their walking trajectory within the next 4–6 steps, non-fallers were able to redirect attention away from the walking path and from people to fixate for longer on the inconsequential but interesting stationary furniture and artwork of the near environment in the lobby. Further, fallers fixated for longer average durations on near people and for shorter average durations on the near environment and far walking path in the real world relative to the lab environment, but there was no similarly significant environmental influence on non-fallers. This environmental influence on fallers, and lack of one on non-fallers, may indicate that fallers were not able to look around or plan as far ahead as non-fallers while walking in the real world environment. Fallers may have been more anxious about looking away from the people around them and in their walking path. Previous research has demonstrated that anxiety in older adults is a negative predictor of successfully avoiding obstacles while walking [38] and is related to longer fixation times on walking targets [39]. Additionally, the fallers in this study performed worse on all three of the executive function and information processing speed tests than the non-fallers (Table 1), which supports that they may have needed more time to process the information and plan their walking trajectory with each new step. A study by Persad et al. [38] provided evidence that, in addition to anxiety, an inability to maintain focused and effortful attention over time as well as selectively attend to relevant stimuli, which are assessed by the three executive function and information processing speed tests utilized in this study [40], were negatively related to successful avoidance of obstacles. The fact that fallers performed worse on all three cognitive tests than non-fallers, but that the covariate, CTMT, did not have a significant effect on any of the gait or gaze variables may support the idea that anxiety, and possibly other factors associated with a fall history, may mediate how age-related declines in cognition impact gaze behavior and planning during walking. Regardless, the fact that fallers did not look as far ahead while walking in a busy environment, relative to a quiet lab environment, may have serious implications for their ability to plan ahead to avoid hazards while walking in their everyday lives. This assertion may be especially important because no similar environmental influence was observed in non-fallers.

After adjusting for baseline gait speed with the covariate TUG, it became apparent that some of the previously mentioned differences in average fixation durations between fallers and non-fallers were a function of differences in gait speed typical of fallers and non-fallers [31], but new group differences also emerged. Specifically, in the lab, fallers fixated for shorter average durations on the near and far walking path and far people than non-fallers, and in the real-world environment, fallers fixated for overall shorter average durations than non-fallers. These results are important because, again, visual information is only acquired and stored in memory during fixations [35], thus they imply that regardless of gait speed, fallers and non-fallers may differ in their strategies of visual information processing in their everyday lives. Taken together with the fact that the fallers’ worse performance than non-fallers on all three of the executive function and information processing speed tests already implies that the fallers in this study may have needed more time than non-fallers to process visual environmental information and plan their walking trajectory with each new step [38], these results provide evidence that fallers may be acquiring and storing less visual information about each of the AOIs they fixate upon than non-fallers. This difference in visual information processing may have serious consequences for walking safety, and visual focus while walking may be a fall-risk factor that warrants further attention. Older adults prone to falling may benefit from eye movement training that teaches them where to look while walking in busy environments and should be the focus of future studies.

This study has a few limitations. First, the study is limited by a sample size that was slightly smaller than the power analysis dictated due to technical difficulties during data collection. However, with 26 rather than 28 participants, the loss of statistical power was minimal, reaching an a priori α = 0.05 and power = 88%; rather, the non-significant results were due to small and unimportant effect sizes. Second, in typical research designs, a real-world problem/situation is recreated in a lab environment that can be tested under highly controlled conditions, but recreating complexity and unpredictability at the same time in a lab setting in an ecologically valid manner would be very difficult to achieve, if possible at all. Using an uncontrolled real-world setting allowed us to better observe more natural everyday behaviors than a controlled laboratory setting would but did limit our ability to completely control the environment across participants. We controlled testing conditions as best as we were able by scheduling data collections for times of day when the hospital was reliably busy, by having participants complete two trials for each test condition to be able to report an average performance and dilute the effect of any random events or chance encounters, and by comparing the average performances of the fallers and non-fallers and in the lab and real-world environments, further diluting any random events or chance encounters. The pedestrian traffic in the hospital lobby was specifically not controlled across participants because not controlling the environmental busyness maximizes the ecological validity of this environment. People in the environment therefore may have looked or behaved differently (e.g., walking at different speeds or wearing different types of clothing) from participant to participant, which could have impacted participant behavior, but we believe the impact of these differences to be negligible when comparing across the two groups. Additionally, although the number of people in the real-world environment varied somewhat from participant to participant, there were no significant differences in environmental busyness between fallers and non-fallers in either of the two environments. Third, instrumenting participants and then observing them as they walked in the real-world environment may have caused them to behave differently than if they were walking in everyday life without being observed or wearing extra equipment. We tried to minimize the impact of the instrumentation on the participants’ behavior by utilizing wireless systems that were lightweight and by placing the equipment on participants as early as possible during each data collection so as to give participants time to become accustomed to each piece before they were given trial instructions and commenced walking. Fourth, near AOIs were defined as within 4–6 steps of the participant, and far AOIs were defined as beyond 4–6 steps, using a range of distances to distinguish between the two categorizations instead of an exact distance (i.e., 6 steps). While allowing participants to freely move their head during each trial prevented using a more exact distance to distinguish between near and far AOIs, it ensured that the gaze data represented realistic head movements and gaze behavior, which we deemed to be the more important consideration. A single rater categorized all of the fixations into near or far AOIs to maximize consistency between trials and participants. Finally, this study focused on spatio-temporal gait parameters and gaze behavior during single-task walking, but the inclusion of a dual-task measure of walking may have provided clearer insight into the automaticity of gait between fallers and non-fallers and the inclusion of a measure of balance control during walking may have provided insight into differences of dynamic control between fallers and non-fallers. With high automaticity, fixating on more interesting stimuli such as people outside of the walking path and artwork of the near environment in the real world would not be surprising but could potentially be detrimental to walking safety if declines in information processing speed and executive function inhibited planning to avoid hazards in the walking path. However, with lower automaticity, fixating on people and the surrounding environment while walking instead of the walking path could be detrimental to walking safety, especially with declines in information processing speed and executive function. Further, the gait measures focused on spatio-temporal parameters recorded from lower limb movement, but it is possible that a measure of dynamic balance control, recorded from the trunk or approximating the center of mass of an individual, could have provided evidence of other important differences between fallers and non-fallers in navigating a real-world environment. These types of analyses are outside of the scope of the current study, but will be explored in future studies.

## Conclusions

This study provides evidence that there is an environmental effect on what older adults visually attend to while walking and that gaze while walking is different between fallers and non-fallers. Specifically, both fallers and non-fallers fixated more frequently on people and less frequently on their walking path and surrounding environment in the real world compared to a low-distraction environment. Perhaps more importantly, there appear to be differences between older adults with a history of falling and older adults without a history of falling, with regards to what they visually attend and for how long. Specifically, in the real world, fallers fixated for shorter durations on their immediate surrounding environment than non-fallers, and only fallers decreased fixation durations on their walking path and increased fixation durations on nearby people from the lab to the real world. There is no similar environmental influence on non-fallers. After adjusting for baseline differences in mobility and cognition, fallers were observed to fixate for generally shorter durations than non-fallers in the real world and the lab. Environmental effects on gait were minimal for both fallers and non-fallers. These results may indicate that visual focus while walking should be further explored as a possible fall-risk factor.

## Supporting information

S1 Data (XLSX)
